# Immediate cortical glial alterations following spinal cord injury: Evidence from a novel in vitro model

**DOI:** 10.1113/EP092809

**Published:** 2025-10-06

**Authors:** Luca Mio, Giulio Pistorio, Atyieh Mohammadshirazi, Giuliano Taccola, Carmen Falcone

**Affiliations:** ^1^ Neuroscience Department International School for Advanced Studies (SISSA) Trieste Italy; ^2^ Applied Neurophysiology and Neuropharmacology Lab Istituto di Medicina Fisica e Riabilitazione (IMFR) Udine Italy; ^3^ Department of Biological Sciences Towson University Towson Maryland USA

**Keywords:** cerebral cortex, glia, motor cortex, remote damage, spinal trauma

## Abstract

Spinal cord injury (SCI) triggers immediate and widespread pathophysiological changes not only at the site of impact but also beyond it, including alterations in remote cortical regions. Here, we report early astrocytic changes in the cerebral cortex following SCI at birth, identified using two specific glial markers in an innovative in vitro model of the entire central nervous system (CNS) isolated from neonatal rats. Immunohistochemical analyses revealed a significant reduction in cortical astrocyte density, first observed in the dorsomedial motor cortex (M1) within 25 min post‐injury, followed by progressive changes in the ventrolateral somatosensory cortex (S1/S2) at 2 h post‐injury. These findings indicate that SCI initiates a rapid and dynamic reorganization of cortical glial networks, shedding new light on astrocytic responses to spinal trauma.

## Abstract (highlights)

- **What is the central question of this study?**
  Do acute remote brain changes occur after traumatic spinal cord injury?
- **What is the main finding and its importance?**
  A significant reduction in astrocyte density in the primary motor cortex is observed as early as 25 min post‐injury, with no notable changes detected at the later time point. Conversely, astrocyte density in the primary and secondary somatosensory cortices remains unchanged in the acute phase but exhibits significant rearrangements 2 h after injury.

## 1 INTRODUCTION

Astrocytes are a class of glial cells in the central nervous system (CNS), playing essential roles in neurodevelopment (Clarke & Barres, 2013; de Majo et al., 2020), synaptic regulation (Lawal et al., 2022; Lyon & Allen, 2022; Tan et al., 2021), metabolic support (Chen et al., 2023; Murat & García‐Cáceres, 2021) and neuroinflammation (Giovannoni & Quintana, 2020). Amongst other functions, these cells contribute to blood–brain barrier integrity, ion homeostasis, neurotransmitter recycling and immune responses. Their ability to respond dynamically to injury or disease makes them key players in neuropathological processes, including spinal cord injury (SCI). Astrocytes undergo structural and functional changes following trauma, a process known as reactive astrogliosis, which can have both neuroprotective and neurotoxic consequences depending on the severity of the injury and the local cellular environment.

A crucial marker for identifying astrocytes is S100β, a calcium‐binding protein highly expressed in these cells. Unlike glial fibrillary acidic protein (GFAP), which primarily labels layer I astrocytes and fibrous astrocytes in the white matter of rodents, S100β stains a broader population of astrocytes across multiple cortical layers (Jurga et al., 2021). Additionally, at early developmental stages, GFAP is expressed in radial glial cells, the progenitors of astrocytes, making it an unreliable marker for mature astrocytes during early postnatal development. In contrast, S100β expression is retained in differentiated astrocytes, making it a more robust indicator for tracking astrocytic changes in both normal and pathological conditions. Another established marker for astrocytes is glutamine synthetase (GS), an enzyme predominantly expressed by astrocytes in the CNS, playing a critical role in glutamate metabolism and ammonia detoxification (Norenberg & Martinez‐Hernandez, 1979). GS converts glutamate into glutamine, thereby regulating neurotransmitter cycling and maintaining glutamate homeostasis, essential for synaptic function and neuronal health (Schousboe et al., 2013). Due to its preferential localization in astrocytes, particularly in mature, non‐reactive cells, it is frequently employed as an astrocyte marker in studies of brain physiology and pathology.

SCI is a devastating condition that not only affects the injury site but also induces secondary changes across the CNS. While neuronal loss and synaptic dysfunction have been well‐documented (Aguilar et al., 2010; Chen et al., 2023; Dahlberg et al., 2018), the impact of SCI on cortical glial cells remains underexplored. Recent evidence suggests that traumatic SCI can lead to reactive gliosis in brain regions, with potential consequences for neural plasticity and recovery (Hu et al., 2022; Wu et al., 2014). However, the precise timeline and regional specificity of these changes remain unclear, especially in paediatric SCIs. Using a novel in vitro preparation of the entire neonatal rat CNS, we investigated the immediate effects of SCI on astrocytes in distinct cortical regions (i.e., dorsomedial motor cortex and ventrolateral somatosensory cortex), aiming to characterize their spatiotemporal dynamics following spinal injury.

## 2 METHODS

All experimental procedures were approved by the Ethics Committee of the International School for Advanced Studies (SISSA) under protocol number 22DAB.N.52M. All procedures were conducted in accordance with the Italian Animal Welfare Act (Legislative Decree 24/03/2014, No. 26), which implements the European Directive 2010/63/EU on the protection of animals used for scientific purposes. In vitro preparations of the entire CNS were obtained from 16 neonatal rats (P0–P3). Surgical procedures were preceded by 7–11 min of cryoanaesthesia at room temperature (Danneman & Mandrell, 1997; Phifer & Terry, 1986; Zimmer et al., 2020). Following the loss of the tail pinch reflex, the forehead was ablated at the orbital line and the skin was removed from the skull and back. The chest and forelimbs were ventrally detached and the preparation was transferred to a Sylgard‐coated Petri dish under a microscope, fully immersed in oxygenated Krebs solution (113 mM NaCl, 4.5 KCl, 1 mM MgCl_2_ 7H_2_O, 2 mM CaCl_2_, 1 mM NaH_2_PO_4_, 25 mM NaHCO_3_, 30 mM glucose; pH 7.4, 298 mOsm/kg, gassed with 95% O_2_/5% CO_2_). Craniotomy and dorsal and ventral laminectomies were then performed to expose the entire CNS, which was isolated from the olfactory bulbs to the cauda equina by transecting all cranial nerves, dorsal and ventral roots (Nicholls et al., 1990).

A controlled mechanical injury was applied to the thoracic (T10) region of the spinal cord using a custom‐engineered micro‐impact device, specifically designed to facilitate simultaneous electrophysiological recordings from the neonatal CNS in vitro (Mohammadshirazi et al., 2023, 2025). This device, currently undergoing patenting at SISSA, is available upon request (https://www.valorisation.sissa.it/device‐mechanically‐stimulating‐biological‐material‐and‐its‐procedure).

To ensure precise targeting, the 2‐mm‐diameter impactor tip was accurately positioned on the ventral spinal cord surface using a micromanipulator. The system was controlled via dedicated software, allowing for precise adjustment of impact parameters, including displacement, speed, acceleration, deceleration and pause time. In our experimental set‐up, a severe non‐transecting injury was induced by driving the impactor tip 2656 µm into the spinal cord at a mean velocity of 4 mm/s, with acceleration and deceleration maintained at 6.1 ± 0.05 mm/s^2^. Following the impact, the tip was retracted to its original position at the same speed and acceleration.

After spinal trauma, brain samples were collected and subsequently processed for immunohistochemistry. Coronal sections (20 µm thick) were obtained using a cryostat and subjected to free‐floating immunohistochemical staining, following a previously published protocol (Ciani et al., 2023).

To reduce autofluorescence, tissue slices were pre‐treated with 0.1% Sudan Black (in 70% ethanol) for 30 min, then briefly rinsed with 70% ethanol before incubation in 10% serum blocking solution for 1 h at room temperature. Sections were then incubated overnight at 4°C with a rabbit anti‐S100β primary antibody (Abcam, Cambridge, UK, cat. no. ab52642, RRID: AB_882426) or a rabbit anti‐GS primary antibody (Abcam cat. no. ab73593, RRID:AB_2247588) diluted in 2% blocking solution.

The next day, sections were washed three times with 1× phosphate buffered saline, followed by incubation with a polyvalent AlexaFluor 488‐conjugated anti‐rabbit secondary antibody (1:400, Thermo Fisher Scientific, Waltham, MA, USA, cat. no. A‐21206, RRID: AB_141633) diluted in 2% serum blocking solution. Finally, nuclei were counterstained using 4′,6‐diamidino‐2‐phenylindole (DAPI, 1:500, Hoffmann‐La Roche, Basel, Switzerland, cat. no. 10236276001) to enable nuclear visualization.

High‐resolution images were captured using a Nikon (Tokyo, Japan) A1/R confocal microscope equipped with a ×60 oil‐immersion objective. To ensure comprehensive imaging of cellular structures, z‐stack acquisitions were performed, consisting of at least 20 steps of 1‐µm increments, covering a minimum optical section thickness of 20 µm.

Immunofluorescence images of the cerebral cortex were analysed to quantify the number of S100β^+^ or GS^+^ astrocytes and DAPI‐labelled total cells in each region of interest (ROI) using Volocity software (Quorum Technologies Inc., Sacramento, CA, USA), by applying standardized intensity‐based thresholds to segmented images, followed by automated counting of positively stained objects within defined regions of interest.

Statistical analyses were conducted using GraphPad InStat 3.10 (GraphPad Software, Boston, MA, USA). Non‐parametric data are presented as median and interquartile range (IQR). Sample sizes are indicated as ‘*n*’, consistently referring to the number of animals used in the experiments. Normality was assessed prior to group comparisons to determine the use of parametric or non‐parametric tests. Statistical analysis was done with the Kruskal–Wallis test followed by pairwise multiple comparisons with Dunn's Method and statistical significance was set as *P* < 0.05.

Data distributions were visualized with box‐and‐whisker plots generated in Microsoft Excel. Boxes represent the IQR, with the median shown as a line. Whiskers extend to the most extreme values within 1.5 times the IQR; outliers beyond this range are plotted individually.

## 3 RESULTS

To assess the impact of spinal injury on brain structures, the cerebral cortex was examined at two distinct time points: the acute phase (25 min post‐injury) and the late phase (2 h post‐injury). Since these experiments were conducted during the peak of astrogenesis in rats, astrocyte density in the cerebral cortex was measured to evaluate the potential effects of the spinal insult. Astrocytes were identified through immunostaining for both S100β and GS markers, followed by DAPI counterstaining. Astrocyte density was quantified as the ratio of S100β and GS‐positive cells to the total cell count.

**FIGURE 1:**
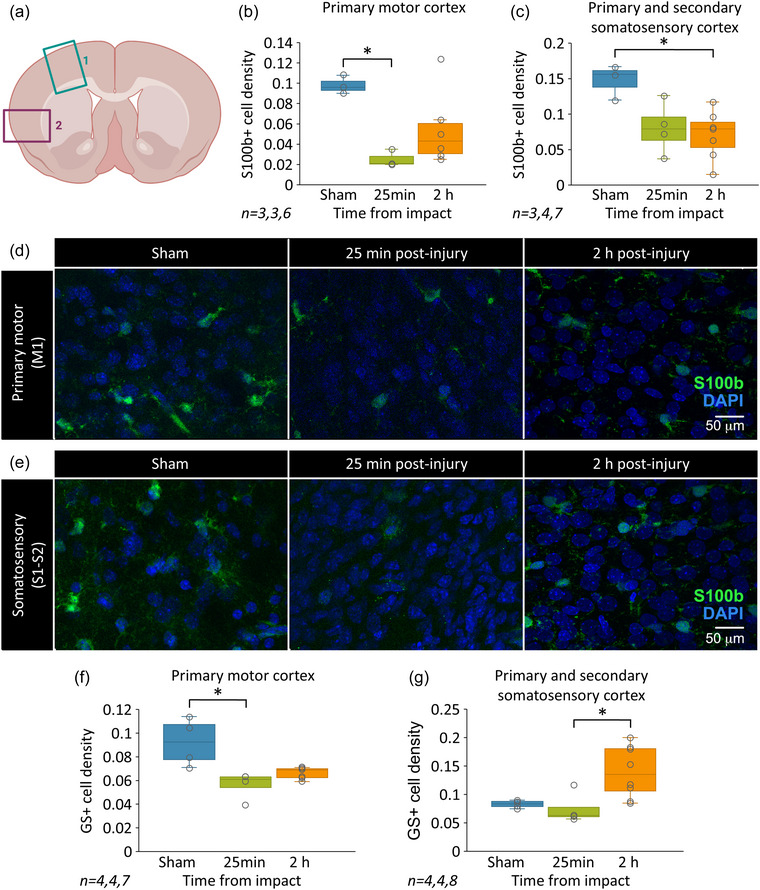
Remote changes in cortical glia occur after spinal damage. (a) Illustration showing a coronal section of the cerebral cortex used for analysis. Rectangle 1 indicates the dorsomedial cortex, including the primary motor area; rectangle 2 indicates dorsolateral and ventrolateral cortices, including both primary and secondary somatosensory cortices. (b) Mean S100β^+^ cell density in the primary motor cortex, 25 min and 2 h post‐injury, compared to sham (*n* = 3, 3, 6). (c) Mean S100β^+^ cell density in the primary and secondary somatosensory cortices, 25 min and 2 h post‐injury, compared to sham (*n* = 3, 4 and 7 for sham, 25 min and 2 h, respectively). (d) Representative confocal images from the primary motor cortex in sham, 25 min and 2 h post‐injury. S100β (in green) was used to label astrocytes and DAPI (in blue) was used to counterstain total cell nuclei. Images were taken at ×60 magnification using an oil objective lens. (e) Confocal images from the somatosensory cortex at the same time points as in (d). (f) Mean GS^+^ cell density in the primary motor cortex, 25 min and 2 h post‐injury, compared to sham (*n* = 4, 4 and 7 for sham, 25 min and 2 h, respectively). (g) Mean GS^+^ cell density in the primary and secondary somatosensory cortices, 25 min and 2 h post‐injury, compared to sham (*n* = 4, 4 and 8 for sham, 25 min and 2 h, respectively).

In the dorsomedial cortex, corresponding to the primary motor area (M1, Figure 1a, green box), S100β^+^ astrocyte density showed a significant reduction as early as 25 min post‐injury (25.85% of sham), with partial recovery by 2 h post‐injury (55.78% of sham; *P* = 0.033, Kruskal–Wallis test; *n* = 3, 3 and 6 animals used for the analysis for sham, 25 min and 2 h, respectively; Figure 1b,d). Conversely, in the ventrolateral cortex, including both primary and secondary somatosensory areas (S1 and S2, Figure 1a, magenta box), S100β^+^ astrocyte density initially remained at 54.35% of sham at 25 min post‐injury but declined significantly to 50.90% of sham by the 2 h mark (*P* = 0.037, Kruskal–Wallis test, *n* = 3, 4, 7, Figure 1c,e).

To evaluate if these results were restricted to the S100β^+^ astrocyte population or were valid for a broader astrocyte population, we repeated a similar quantification by using another well‐known astrocyte marker, GS. Similarly to the quantification of the S100β marker, GS^+^ astrocyte density in M1 was significantly reduced 25 min post‐injury (60.54% of sham; *P* = 0.0087, Kruskal–Wallis test; *n* = 4, 4 and 7 animals used for the analysis for sham, 25 min and 2 h, respectively; Figure 1f). In contrast, in S1 and S2 regions, GS^+^ astrocyte density significantly increased 2 h post‐injury (183.94% of sham; *P* = 0.0158, Kruskal–Wallis test; *n* = 4, 4 and 8 animals used for the analysis for sham, 25 min and 2 h, respectively; Figure 1g). Notably, in the S1/S2 areas, GS^+^ astrocyte density was unaffected at 25 min post‐injury (90.36% of sham).

## 4 DISCUSSION

Our findings revealed a significant reduction in the density of both S100β^+^ and GS^+^ astrocyte subpopulations in the primary motor cortex as early as 25 min post‐injury, with no notable changes detected at the later time point (2 h post‐injury). Conversely, S100β^+^ and GS^+^ astrocyte density in the primary and secondary somatosensory cortices remained unchanged in the acute phase but exhibited significant changes 2 h after injury. This pattern of glial response closely mirrors the spreading depolarization from the spinal injury site as reported in a previous finding (Mohammadshirazi et al., 2025), highlighting the rapid and dynamic nature of cortical astrocyte alterations following SCI. While the local effects of SCI have been extensively characterized in various models, its broader impact on the cerebral cortex, remains less well understood. Previous studies have shown that SCI can induce both immediate and long‐term reorganization within the cerebral cortex. For instance, anaesthesia‐dependent functional reorganization of the primary somatosensory cortex was reported in rats following a complete thoracic spinal cord transection (Aguilar et al., 2010). However, our results were not confounded by the effect of anaesthesia, as our experimental model did not include in vivo procedures requiring anaesthetic administration. Furthermore, SCI has been associated with neuroinflammation in the brain, particularly within the primary motor cortex, where it leads to microglial activation (Hu et al., 2022; Wu et al., 2014). Astrocytes play a key role in these processes, contributing to neural circuit remodelling, regulating synaptic development and function, and participating in neuroinflammatory responses. Their actions can be either neuroprotective or neurotoxic, depending on the injury context. However, to the best of our knowledge, the immediate impact of SCI on cortical astroglia has not been previously explored. Our study aimed to assess acute astrocytic responses in the primary motor cortex and the primary and secondary somatosensory areas following SCI at birth.

Given that our experiments were conducted during the peak of cortical astrogenesis in neonatal rodents (P0–P3), these astrocytic changes may reflect alterations in the rate of astrocyte generation rather than a direct loss of astrocytes. However, considering the pronounced and rapid decrease in S100β^+^ and GS^+^ astrocyte density shortly after SCI, alternative explanations must be acknowledged. One possibility is that SCI selectively affects the expression levels of S100β, a calcium‐binding protein typically associated with a subset of mature astrocytes, rather than causing astrocyte loss. In line with this, we concluded that the observed reduction may indeed reflect a specific downregulation of S100β and GS expression or, at least, of a more mature astrocyte subpopulation. Additionally, while our quantification approach using Volocity software involved intensity‐based thresholds designed to accurately segment and distinguish astrocytic cell bodies from branches or smaller fragments, we acknowledge the inherent challenges in completely excluding all non‐cell‐body structures Thus, we propose that the observed reduction in S100β^+^ and GS^+^ astrocyte density likely results from a SCI‐induced modulation of astrocytic protein expression or altered cortical astrogenesis dynamics rather than extensive cell death, underscoring the complex and widespread effects of SCI on cortical development and function beyond the immediate injury site. Interestingly, not all of the findings obtained using the S100β marker were recapitulated when looking at the GS^+^ astrocyte population. GS is a marker broadly expressed across astrocyte types and is pivotal for their role in glutamate homeostasis, while S100β expression has often been primarily associated with protoplasmic astrocytes within the grey matter. The differential trend between GS and S100β observed in the somatosensory areas 2 h after injury suggests a nuanced astrocytic response, potentially reflecting distinct functional states or adaptive metabolic responses in astrocytes following SCI. Exploring the biological mechanisms underlying these opposing trends could provide important insights into the specificity of astrocyte subpopulation responses and their functional roles in early cortical remodelling after spinal trauma.

Our findings highlight a complex interplay between SCI at birth and cortical astrocyte responses, with distinct spatial and temporal patterns of astrocyte alterations across different cortical regions. The observed alterations in astrocyte density could have long‐term consequences for cortical plasticity and functional recovery. Future studies should aim to identify the molecular mechanisms driving these astrocytic changes following SCI, including potential variations in response to injuries of different severities, and to compare these effects in both neonatal and adult models. Investigating the signalling pathways underlying astrocyte regulation post‐SCI could provide critical insights for developing targeted interventions to assess and modulate astrocyte activity in the context of injury and recovery. Additionally, exploring the interactions between astrocytes, other glial cells, and neurons in response to SCI could further elucidate the role of astrocytes in cortical reorganization and functional outcomes. For example, it is still unknown whether immediate pathological signals are transiently triggered in the brain right after an impact to the spinal cord. The presence of pathological signs (such as the overexpression of pStat3 – often upregulated in reactive astrocytes in the context of neuroinflammation or neuropathology) could actually provide a novel marker to more realistically characterize the severity of a lesion and envisage potential recoveries.

## AUTHOR CONTRIBUTIONS

Giuliano Taccola and Carmen Falcone contributed to the study conception and design. Luca Mio, Giulio Pistorio and Atyieh Mohammadshirazi performed acquisition and analysis of data for the work. Giulio Pistorio and Atyieh Mohammadshirazi prepared the figures. The manuscript was drafted by Giuliano Taccola and Carmen Falcone and critically commented on by all authors. All authors approved the final version of the manuscript. All authors agreed to be accountable for all aspects of the work in ensuring that questions related to the accuracy or integrity of any part of the work are appropriately investigated and resolved. All persons designated as authors qualify for authorship, and all those who qualify for authorship are listed.

## CONFLICT OF INTEREST

The authors have no relevant financial or non‐financial interest to disclose. The impactor adopted in the study is currently being patented by SISSA and is available upon request.

## Data Availability

The datasets generated during and/or analysed during the current study are available from the corresponding author on reasonable request.
